# Predictive biomarkers for cancer immunotherapy with immune checkpoint inhibitors

**DOI:** 10.1186/s40364-020-00209-0

**Published:** 2020-08-26

**Authors:** Rilan Bai, Zheng Lv, Dongsheng Xu, Jiuwei Cui

**Affiliations:** Cancer Center, the First Hospital of Jilin University, 71 Xinmin Street, Changchun, Jilin, 130021 China

**Keywords:** Neoplasm, Immune checkpoint inhibitor, Predictive biomarker, Tumor mutation burden, Programmed death ligand-1

## Abstract

Although the clinical development of immune checkpoint inhibitors (ICIs) therapy has ushered in a new era of anti-tumor therapy, with sustained responses and significant survival advantages observed in multiple tumors, most patients do not benefit. Therefore, more and more attention has been paid to the identification and development of predictive biomarkers for the response of ICIs, and more in-depth and comprehensive understanding has been continuously explored in recent years. Predictive markers of ICIs efficacy have been gradually explored from the expression of intermolecular interactions within tumor cells to the expression of various molecules and cells in tumor microenvironment, and been extended to the exploration of circulating and host systemic markers. With the development of high-throughput sequencing and microarray technology, a variety of biomarker strategies have been deeply explored and gradually achieved the process from the identification of single marker to the development of multifactorial synergistic predictive markers. Comprehensive predictive-models developed by integrating different types of data based on different components of tumor-host interactions is the direction of future research and will have a profound impact in the field of precision immuno-oncology. In this review, we deeply analyze the exploration course and research progress of predictive biomarkers as an adjunctive tool to tumor immunotherapy in effectively identifying the efficacy of ICIs, and discuss their future directions in achieving precision immuno-oncology.

## Background

Immune checkpoint inhibitors (ICIs) therapy has ushered in a new era of anti-tumor therapy, with sustained responses and significant survival advantages observed in multiple tumors. Anti-programmed cell death-1/programmed cell death-ligand 1 (PD-1/PD-L1) antibody has been approved for second-line or first-line treatment in a variety of malignant neoplasms, including melanoma, lung cancer, renal cell carcinoma (RCC), head and neck squamous cell carcinoma (HNSCC) and gastroesophageal cancer [1, 2]. However, despite the breakthrough in clinical treatment with ICIs, most patients do not benefit. Pembrolizumab or nivolumab has an objective response rate (ORR) of 40–45% in first-line melanoma and 20% in second-line non-small cell lung cancer (NSCLC) [3–5]. Therefore, in recent years, more and more attentions have been paid to the identification and development of predictive biomarkers for the efficacy of ICIs, and more in-depth and comprehensive understanding has also been obtained in recent years, including new data on biomarkers of tumor genome and neoantigen, tumor immune microenvironment phenotype, liquid biopsy biomarkers, host-related factors and all of which have made many new advances in the corresponding fields. With the development and continuous improvement of multiplex immunohistochemical technology, high-throughput sequencing and microarray technology, a variety of biomarker strategies have emerged and gradually realized the process from the identification of single marker to the development of multifactorial synergistic predictive markers. The development of predictive biomarkers contributes to revealing the therapeutic mechanisms of ICIs and the interaction mechanisms between tumor and host immunity, achieving decision-making of individualized anti-tumor immunotherapy, monitoring efficacy and disease development, guiding clinical trial design, as well as for further understanding of drug resistance mechanisms and tumor prognosis. In this review, we deeply analyze the exploration course and research progress of predictive biomarkers as an adjunctive tool to tumor immunotherapy in effectively identifying the efficacy of ICIs. It should be pointed out here that when reading and collating, we try to read and include all the relevant articles. In the process of selecting articles, we include the authoritative articles published in high-level journals or the latest research results, and objectively describe and analyze their roles in this field, as well as discuss the reasons that different research results may be involved.

## Advances of multiple predictive biomarkers to ICIs efficacy

(i). **Tumor genome and neoantigen biomarkers**

### Tumor mutation burden

Significant correlations between high tumor mutation burden (TMB) and response to ICIs have been reported in several cancer types [6], including urothelial carcinoma [7], small cell lung cancer (SCLC) [8], NSCLC [9–11], melanoma [12], and human papilloma virus (HPV)-negative HNSCC [13]. A meta-analysis of 27 cancer types showed that the mean response rate was positively correlated with log (TMB) [14]. The National Comprehensive Cancer Network (NCCN) guidelines have adopted TMB as the recommended test for patients with NSCLC receiving immunotherapy. Although the results in some clinical studies of RCC [15], HPV-positive HNSCC [13], and melanoma receiving anti-PD-1 after recurrence [16] showed that TMB alone also did not clearly distinguish responders and predict OS, it is still exciting that multiple studies in the 2020 American Society of Clinical Oncology (ASCO) meeting have confirmed the predictive value of TMB in immunization or combination therapy (KEYNOTE-061 study [17, 18], CONDOR study [19], EAGLE study [20], EPOC1704 study [21], etc.), consolidating its status of TMB as an independent predictor. And in April 2020, the U.S. Food and Drug Administration (FDA) prioritized the approval of TMB as a companion diagnostic biomarker for pembrolizumab.

Nonetheless, the cut-off values of TMB were defined differently across studies and assay platforms, such as atezolizumab > 16 mt/Mb in urothelial cancer, pembrolizumab > 23.1 mt/Mb in NSCLC, and atezolizumab ≥13.5, ≥15.8, or ≥ 17.1 mt/Mb in NSCLC [22–25], and nivolumab plus ipilimumab ≥10 mt/Mb in NSCLC [10, 26], which needs further study to confirm the optimal cut-off value in different tumors. Moreover, the NGS panels have approved by the FDA that can be used to estimate TMB include the MSK-IMPACT and FoundationOne CDx panel, the detection results of which are highly consistent with whole exome sequencing (WES) [11, 27], and other solutions are under development. A study detecting TMB (cut-off value at 20 mt/Mb) in 4064 NSCLC patients with the FoundationOne platform containing a 395 gene panel found that compared with TMB-L patients, overall survival (OS) and DCR was significantly improved in TMB-H patients treated with anti-PD-1/L1 drug [11]. Both WES and targeted NGS (a 422-cancer-gene panel) performed in 78 patients with NSCLC treated with anti-PD-1/L1 demonstrated that TMB-H population has a significantly better durable clinical benefit (DCB) and progression-free survival (PFS) [27]. These findings demonstrate the feasibility of comprehensive genomic profiling (CGP), but the design of optimal next generation sequencing (NGS) panel that is more accurate, comprehensive and cost-effective is still not clear. In addition, given that bTMB was identified as a predictor of PFS but failed to differentiate patients with OS benefits, researchers consider the need to explore other more precise factors, e.g. allele frequency (AF). A study that developed a new bTMB algorithm in two independent cohorts (POPLAR and OAK) showed that modified bTMB, low AF bTMB (LAF-bTMB, mutation counts with an AF < 5%), was significantly associated with favorable (HR = 0.70, 95%CI 0.52–0.95, *p* = 0.02), PFS (HR = 0.62, 95%CI 0.47–0.80, *p* < 0.001), and ORR (p < 0.001) after immunotherapy, but required to be prospectively validated [28]. Finally, static biomarkers are insufficient to accurately predict response due to the complexity of tumor-immune interactions. A recent analysis of tumor genome-wide dynamic detection in pretreatment and on-treatment melanomas found that pretreatment TMB was only associated with OS in untreated patients, while early (4-week) on-treatment change in TMB (ΔTMB) was strongly associated with anti-PD-1 response and OS in the entire cohort [16]. The detection of ΔTMB is helpful for early evaluating the response to therapy of patient, but its clinical usability limited by the difficulty in obtaining tissue samples and high price, while liquid biopsy discussed below might better.

In addition, epigenetic changes are associated with TMB. The latest study investigated the association between TMB and DNA methylation (DNAm) to explore potential complimentary biomarkers for NSCLC immunotherapies. The results showed that high TMB NSCLCs had more DNAm aberrance and copy number variations (CNVs), showing certain value in predicting efficacy, such as HOX gene methylation status and TMB [29] Thus, the correlated exploration of epigenetics has attracted more attention in recent years, and liquid biopsy-based epigenetic studies may become a future research direction. Exploration in Chinese NSCLC patients showed that NSCLCs with high TMB had DNAm aberrance and CNVs. Some insertion and deletion (indel) mutations can lead to frameshifts and more immunogenic neoantigens [30]. In the pan-cancer analysis of 19 cancer types evaluated in The Cancer Genome Atlas (TCGA), RCC had the highest indel mutation load, and frameshift indel mutations were found to produce three times more candidate neoantigens per mutation than nonsynonymous single nucleotide variants (nsSNVs) [30]. Somatic copy number alterations (SCNAs) are another feature of the genomic landscape of tumors, and pan-cancer TCGA analysis revealed an inverse correlation between SCNAs at the single-arm or whole chromosome-level and immune infiltration in 10 tumor types tested [31], and this result was subsequently replicated in a larger study of TCGA [32].

### DNA damage response pathways

Genetic variation involved in DNA mismatch repair (MMR) pathway can lead to microsatellite instability (MSI), a specific type of high TMB tumors, and increased numbers of CD8^+^ tumor infiltrating lymphocytes (TILs), PD-1^+^TILs, and indoleamine 2,3-dioxygenase (IDO)^+^ tumor cells have been shown in MMR deficiency (dMMR) colorectal cancer [33]. Recently, five clinical trials (Keynote-016, 164, 012, 028, 158) including multiple tumor types have shown that patients with dMMR/MSI-H can achieve durable responses to pembrolizmab. Based on this, pembrolizumab is approved by the U.S. FDA for the treatment of any advanced solid tumor with dMMR/MSI-H, and nivolumab in combination with ipilimumab has also shown promising response in dMMR/MSI-H colorectal cancer [34]. In addition, dMMR can also cause mutations in the DNA polymerase gene epsilon/delta 1 (POLE/POLD1), increasing the mutation load and neoantigen load. Analysis of POLE/POLD1 mutations in 47,721 patients with different cancer types showed that patients with these mutations had significantly higher TMB and OS. Therefore, it may be an independent risk factor and prognostic marker for identifying patients who benefit from ICIs [35]. In addition, pathways of base excision repair (BER), homologous recombination repair (HRR), MMR in the DNA damage response (DDR) signaling network contribute more significantly to TMB or neoantigens, which have the highest levels when co-mutated [36]. It had been identified that co-mutations in the DDR pathways of HRR and MMR or HRR and BER, defined as co-mut^+^, are associated with increased levels of TMB, neoantigen load and immune gene expression signatures. Co-mut^+^ patients showed a higher ORR and longer PFS or OS, indicating that co-mut can be used as predictors of response to ICIs and provide a potentially convenient method for future clinical practice [36].

### Specific mutated gene pathways in tumor cells

It is worth noting that alterations of signaling pathways in tumor cells affect the responsiveness to immunotherapy. Patients with mutations in the interferon (IFN)-γ pathway genes, IFNGR1/2, JAK1/2, and IRF1, are poorly responsive to ICIs treatment and confer resistance [37]. A study found that in patients receiving immunotherapy, tumor cells can downregulate or alter IFN-γ signaling pathways such as loss-of-function alleles of genes encoding for JAK1/2, and changes in STAT1 to escape the influence of IFN-γ [38], resulting in poor efficacy and resistance. Recent studies suggest that inactivating mutations in a mammalian analog of the chromatin remodeling SWI/SNF complex and unique genes of the PBAF complex (Pbrm1, Arid2, and Brd7) lead to sensitivities to ICIs [39, 40]. Loss of function of the PBAF complex increased chromatin accessibility to transcription regulator elements of IFN-γ–inducible genes within tumor cells, and subsequently increased production of CXCL9/CXCL10 chemokines, leading to more efficient recruitment of effector T cells into tumors [41]. In human cancers, expression of Arid2 and Pbrm1 are related to expression of T cell cytotoxicity genes, which confirmed in Pbrm1-deficient murine melanomas with strongly infiltrated by cytotoxic T cells and responsive to immunotherapy [15, 41]. In addition, double-stranded RNA (dsRNA) editing enzyme adenosine deaminase acting on RNA (ADAR1) protein can block the IFN-γ signaling pathway and lead to poor ICIs efficacy and resistance. Loss of function of ADAR1 in tumor cells can reduce A-to-I editing of interferon-inducible RNA species and lead to dsRNA ligand sensing by PKR and melanoma differentiation-associated protein 5 (MDA5). This results in growth inhibition and tumor inflammation, respectively, and profoundly sensitizes tumors to immunotherapy [42]. Finally, demethylation positively regulates the transcriptional activity of some immune-related genes, including PD-L1 and IFN signaling pathway genes, sensitizing it to anti-cytotoxic T-lymphocyte-associated protein-4 (CTLA-4) therapy [43].

In addition to the IFN-γ-related signaling pathway, alterations in other tumor genome, such as tumor oncogenes and suppressor genes pathways, and pathways related to tumor cell proliferation and infiltration, can also affect immunotherapy efficacy. Epidermal growth factor receptor (EGFR) and anaplastic lymphoma kinase (ALK) mutations have been shown to be associated with reduced response rates to ICIs and low TMB, and therefore the FDA does not recommend first-line ICIs-treatment in patients with EGRF or ALK positive tumors [44, 45]; certain types of mutations in MDM2/MDM4 and ARID1A can predict non-response to ICIs in high TMB tumors [46]; NSCLC with KRAS and STK11 co-mutated was associated with reduced response and shorter survival in three independent cohorts of patients treated with anti-PD-1 therapy [47], and STK11 deficiency was an independent indicator of poor anti-PD-1 response in NSCLC with KRAS mutant; however, at the 2020 American Association for Cancer Research (AACR) meeting, 33.7% of patients in the Keynote-042 study (NCT02220894) update data were tested for STK11 and KEAP1, and the results showed that patients could benefit from pembrolizumab regardless of STK11 and KEAP1 status, but patients with STK11 mutations did not respond well to chemotherapy, but given that only 1/3 of all patients had mutation detection, the results may be affected; in initial data from studies using targeted NGS panels suggested that duration of ICIs-treatment was associated with certain BRAF and MET alterations, but not TMB status [48]. NOTCH signaling pathway is associated with the occurrence, development and prognosis of tumors, especially with the biological function of cancer stem cells. Recent breakthrough findings have distinguished deleterious NOTCH mutation, showing that it can be used as a potential predictor of favorable ICI response in NSCLC, potentially via greater transcription of genes related to DNA damage response and immune activation [49]. Another tumor-specific inheritance that may influence ICIs efficacy is the aberrant expression of endogenous retroviruses (ERVs). Pan-cancer analysis identified a positive correlation of transcript expression of ERVs with T-cell activity in various tumors [50] and patient prognosis [51]. Furthermore, with the improvement of precision detection technology, the accurate analysis of negative mutation sites helps to identify the possibly effective ones. For example, the analysis of study data of second-line PD-1/L1 inhibitor therapy found that the mPFS of patients with KRAS G12C or G12V was significantly better than that of patients with KRAS mutations at other sites [52].

In addition, several pan-cancer biomarkers are recently approved by the FDA. For example, given the effective ORR of 35.5% and a disease control rate (DCR) of 82% in second-line cholangiocarcinoma patients treated with pemigatinib, a new targeted therapy, the recent FDA approval of pemigatinib for the treatment of previously treated patients with locally advanced or metastatic cholangiocarcinoma with fibroblast growth factor receptor 2 (FGFR2) fusion or rearrangement, and the comprehensive genomic analysis assay, FoundationOne CDx, developed by Foundation Medicine as a companion diagnostic. Also exciting is the recent FDA approval of the targeted anticancer drug capmatinib for the treatment of metastatic NSCLC with MET exon 14 skipping (METex14) mutations, including first-line patients and previously treated patients, also using FoundationOne CDx as a companion diagnostic to help detect specific mutations present in tumor tissue.

### Neoantigen load

Neoantigen load, the number of mutations actually targeted by T cells, may be directly related to the response to ICIs [53–55]. A retrospective study showed that clonal neoantigen burden was associated with the longer OS in primary lung adenocarcinomas (*p* = 0.025) [53]. Traditionally, computational neoantigen predictions have focused on major histocompatibility complex (MHC) binding of peptides based on anchor residue identities, however, neoantigen loads identified by this method are generally not superior to overall TMB in predicting ICIs efficacy or survival [56]. In recent practice, this neoantigen can be assessed by the difference in predicted MHC-I binding affinity between the wild-type peptide and the corresponding mutant peptide, known as the differential agretopicity index (DAI), reflecting clinically relevant immunogenicity of tumor peptide [57]. A high DAI value indicates that the mutant peptide significantly increases binding affinity to MHC compared to the wild-type sequence and can generate more immune responses. Studies on previously published cohorts treated with three ICIs have shown that DAI outperforms TMB and the traditionally defined neoantigen load in predicting survival [58, 59]. In addition, low neoantigen intratumour heterogeneity might also be important for ICIs response. Analysis of the lung adenocarcinoma TCGA database found that combining high mutational load and low intratumoral neoantigen heterogeneity (< 1%) was significantly associated with OS and longer lasting clinical benefit than either variable alone [53]. Another reported method for assessing neoantigen foreignness is based on sequence homology of experimentally validated immunogenic microbial epitopes in the Immune Epitope Database (IEDB) [60], but it does not account for all possible human leukocyte antigen (HLA) contexts. In addition, the detection for neoantigen can be reflected from different levels such as peptides or genomes. A study developed the Neopepsee algorithm using a machine learning approach incorporating integration of nine immunogenicity features and gene mutation expression levels [61], and its application to melanoma and leukemia patients could improve the sensitivity and specificity of neoantigen prediction. Recently it has also been shown that promoter hypermethylation of neoantigen genes may be an important mechanism for immune editing and tumor immune evasion [62], indicating that combined detection of tumor genome and epigenetics may provide more information for immunotherapy efficacy.

(ii). **Tumor immune microenvironment phenotype biomarkers**

### PD-L1 expression

Given that multiple studies in a variety of tumors have demonstrated a positive correlation between PD-L1 expression and response to ICIs or OS, even in first-line combination therapy [63–65], pembrolizumab is currently approved by the FDA for use in patients with PD-L1^+^ (PD-L1 ≥ 50% of tumor cells in first-line treatment and ≥ 1% in second-line treatment) NSCLC and PD-L1 immunohistochemistry (IHC) as a companion diagnostic for anti-PD-1 therapy in NSCLC patients [66, 67]. However, some studies have not detected a significant correlation between PD-L1 expression and response to ICIs [5, 13, 68], and PD-L1 negative patients can still benefit clinically with treatment with ICI or combination treatment with ICIs [69], with ORRs ranging from 11 to 20%. Therefore, PD-L1 cannot yet be a comprehensive and independent biomarker in clinical practice in assessing efficacy, with following challenges still existing. Firstly, PD-L1 assay and antibody are not standardized [70]. Secondly, PD-L1 expression is temporally and spatially heterogeneous [71]. A study of 398 metastatic NSCLC treated with ICIs showed that PD-L1 varies substantially across different anatomic sites and during clinical course, being highest in adrenal, liver and lymph node metastases and lower in bone and brain metastases. And the predictive value of PD-L1 at different biopsy sites for the benefit of ICIs in NSCLC may vary: higher PD-L1 in lung or distant metastasis specimens was significantly associated with higher response rate, PFS and OS, while PD-L1 in lymph node metastasis biopsy was not associated with either response or survival [72]. Thirdly, positive score and cut-off value of PD-L1 expression is not standardized [71]. At present, PD-L1 positive score mainly focuses on the PD-L1 expression level of tumor cells, that is, tumor proportion score (TPS). But PD-L1 is also expressed on immune cells such as lymphocytes and macrophages and stromal cells, thus the investigators introduce the concept of combined positive score (CPS), which is the proportion score of the sum of PD-L1 expressed by tumor cells and tumor-associated immune cells. In addition, PD-L1 expression on immune cells is also considered separately as one of the biomarkers to distinguish the benefit population, called immune positive score (IPS). Herbst et al. [73] showed that response to atezolizumab treatment was significantly associated with high levels of PD-L1 expression on the surface of TILs before treatment, but not with PD-L1 expression on tumor cells (*p* = 0.079). Finally, other inhibitory immune pathways may affect the response to ICIs therapy, including T cell immunoglobulin-3 (TIM-3), lymphocyte activation gene-3 (LAG-3), and V-domain Ig suppressor of T-cell activation (VISTA), which can be used as potential biomarkers for ICIs response.

### Biomarkers of tumor-infiltrating immune cells

#### Overall immune status of tumor microenvironment

The pattern of tumor immune infiltration can be broadly classified into immune-inflamed, immune-excluded and immune-desert [74]. Immune-inflamed is characterized by the presence of CD8^+^ and CD4^+^ T cells in the tumor parenchyma accompanied by the expression of immune checkpoint molecules [75], indicating a potential anti-tumor immune response to ICIs treatment [73]; immune-excluded is characterized by the presence of different immune cell types in the aggressive margin or stroma of tumor, but cannot infiltration into tumor parenchyma [74, 76]. Analysis of pre-treatment samples for anti-PD-1/PD-L1 revealed a relatively high abundance of CD8^+^T cells at the invasive margin in responders, and serial sampling during treatment showed an increased infiltration of CD8^+^T cells into tumor parenchyma [77]; while immune-desert phenotype is characterized by the absence of abundant T cells in the parenchyma or stroma of tumors and poor response to ICI-treatment [73]. Recently, immunoscore has been proposed as a valid marker for characterizing the immune status of tumor microenvironment (TME), classifying tumors, as well as predicting treatment response and prognosis [78], which involves the density of two lymphocyte populations (CD8^+^ and memory [CD45RO^+^] T cells) in the center and invading margin of tumor [79]. Mlecnik et al. [80] evaluated immunoscore in 599 specimens of stage I–IV colorectal tumor and confirmed that it was significantly associated with PFS, DFS, and OS, and multivariate analysis also showed the superiority of immunoscore in predicting disease recurrence and survival. The value of immunoscore to predicting ICIs efficacy is being validated internationally in clinical trials of melanoma and NSCLC [78].

A wider assessment of active immune responses within TME by immune gene expression profiling might effectively predict clinical benefit to ICIs strategies. Analysis of total RNA and genes that were substantially different between the patient groups in 50 pretreatment tumor biopsies revealed at least a 2.5-fold increase in the expression of 22 immune-related genes in clinically active patients, including cytotoxic T cell markers (e.g., CD8A, perforin 1, granzyme B), Th1 cytokines or chemokines, MHC-II, and other immune-related genes (e.g., NKG7, IDO1) [81]. Ascierto et al. [82] screened more than 299 immune-related genes in patients with recurrent breast cancer 1–5 years after treatment and those without recurrence more than 7 years later, and found that five genes (IGK, GBP1, STAT1, IGLL5, and OCLN) were highly overexpressed in patients with recurrence-free survival. In addition, IFN-γ-induced immune gene signatures may be effective biomarkers for predicting the clinical benefit of treatment with ICIs. The study developed IFN-γ scores combining multiple immune variables based on 10 gene signatures, which were then extended to 28 gene signatures in a validation set of 62 melanoma patients, including genes encoding IFN-γ, granzymes A/B, perforin 1, IDO1, and other immune-related genes. Both gene scores showed significant associations with best overall response rate and PFS. Optimized cut-off values for IFN-γ scores based on receiver operating characteristic curve (ROC curve) can achieve a positive predictive value of 59% for responders and a negative predictive value of 90% for non-responders [83].

#### Immune cells with specific phenotypes in TME

The phenotype of TILs also influences the efficacy of ICIs. The study used single-cell mRNA sequencing (scRNA-seq) data analysis to identify two major CD8^+^T cell phenotypes within melanoma: memory-like and exhausted [84], the proportion of which is strongly correlated with response to ICIs. The research further found that the transcription factor 7 (TCF7) is selectively expressed in memory-like T cells, so the ratio of CD8^+^TCF7^+^ to CD8^+^TCF7-TILs is strongly correlated with improved response and survival in melanoma patients treated with anti-PD-1 [84]. Balatoni et al. [85] found that 7 of 11 immune cells in TME were positively associated with OS after treatment, including CD4^+^ and CD8^+^ T cells, FOXP3^+^ T cells, CD20^+^ B cells, CD134^+^ and CD137^+^ cells, and NKp46^+^ cells, and different immune cells at different sites were differently associated with clinical outcomes. Researchers found that only a small proportion of CD8^+^ TILs in tumors could recognize tumor mutation-associated antigens, while another population (bystander cells) was insensitive, and differential CD39 expression was the key molecule that distinguished the two populations [86]. Analysis of peripheral blood from a patient with colorectal cancer who responded rapidly to pembrolizumab treatment showed high expression of CD39 on CD8^+^ TILs, indicating that CD39^+^CD8^+^TIL may be a promising predictive biomarker [86]. The fact of very low level of CD39 expression on CD8^+^TILs in 50% of EGFR-mutant NSCLC is consistent with their low response rate to anti-PD-1 immunotherapy.

In addition, a study showed that Fc domain glycan of the drug and Fcγ receptor (FcγR) expressed by the host bone marrow cells could determine the ability of PD-1- tumor-associated macrophages (TAMs) to capture anti-PD-1 drugs from the surface of T cells, which leads to PD-1 inhibitor resistance [87], and the association of TAMs and poor anti-PD-1 response was reported in melanoma cohorts [88]; anti-PD-1 response was associated with an increase in CD8^+^T cells and natural killer cells (NK cells) and a decrease in macrophages [16]; and high intratumoral myeloid markers were associated with a nearly 6-fold decrease in mPFS after anti-PD-L1 therapy in RCC, emphasizing the inhibitory role of myeloid cells in response to ICIs [89]. In conclusion, immune cells in TME show a great promise in the development of predictive biomarkers for ICIs.

#### Diversity of immune repertoires in TME

Effective T cell responses involve the activation and expansion of specific antigen-reactive T cell clones, so diversity of immune repertoire in intratumoral or peripheral may correlate with ICIs responses and can be quantified as richness and clonality [16]. However, the results seem to be complex, with some studies finding a positive correlation between TIL clonality and the response to ICIs before [90] or after [91] treatment, while others showing that only an increase in TIL clonality during treatment is associated with the response to anti-PD-1 [16, 92]; others show that intratumoral T cell clonality is not associated with survival, while peripheral T cell clonality is inversely associated with PFS and OS [93]. Tumeh et al. [77] further investigated whether baseline TILs have a narrow T cell receptor (TCR) repertoire, focusing on tumor-specific immune responses and whether this narrow TCR repertoire correlates with pembrolizumab responses. They found that responding patient had more restricted usage of the TCR beta chain (ie, a more clonal, less diverse population) than patients with progressive disease, and showed a 10-times increase in these clones after treatment, implying a tumor-specific response to treatment in these patients. Notably, baseline TCR clonality was not highly correlated with TIL density, suggesting that some patients with restricted TCR clonality specific for tumor antigens may still benefit from anti-PD-1 therapy even though TIL density is low. Recently, researchers have proposed the immune repertoire (IR)-Index, the average frequency of shared TCR clones in T clones in TILs and peripheral PD-1^+^CD8^+^ T cells. They found that neoantigen-stimulated TCR agreed with IR-Index, and patients with high IR-index had better immune activation and higher gene expression profiles (GEPs) score, subsequently they confirmed the predictive value of IR-index to ICIs efficacy (DCR/PFS). But considering that it is difficult to sort out PD-1^+^CD8^+^ T cells in tumor tissue, based on two separate patient cohorts, a research confirmed that TCR repertoire diversity and clonality of peripheral PD-1^+^CD8^+^T cells may serve as noninvasive predictors of clinical outcomes after ICIs in patients with NSCLC [94]. The viewpoints of T cell diversity and TCR clonality as markers of ICIs efficacy need to be further validated in a large patient population.

(iii). **Liquid biopsy biomarkers**

### Peripheral blood cell biomarkers

Peripheral blood is a non-invasive source to explore potential biomarkers for ICIs, and although associations with clinical benefit and survival have been observed, its effectiveness has not been validated in prospective studies. Analysis of melanoma treated with ipilimumab showed that improved OS and PFS were associated with baseline values of peripheral blood components, including low absolute neutrophil count, low neutrophil-to-lymphocyte ratio (NLR), low absolute monocyte count, low frequency of myelogenous suppressor cells, high frequency of FoxP3^+^ Treg cells, high lymphocyte frequency, high eosinophil count; and clinical benefit also associated with the dynamic changes of blood markers during treatment, including decreased FoxP3^+^Treg concentrations and increased lymphocyte and eosinophil counts [95]. Reports in patients with melanoma treated with pembrolizumab and in patients with NSCLC treated with nivolumab have shown that NLR is associated with worse tumor response [96, 97]. Multivariate analysis in melanoma patients treated with anti-PD-1 antibodies showed that NLR was the only factor associated with worse ORR and shorter PFS, indicating that NLR is a strong predictor of worse outcome in patients treated with ICI [96]. Low baseline lactate dehydrogenase (LDH) levels, high relative/absolute eosinophil counts, and relative lymphocyte counts were associated with prolonged OS in anti-PD-1 and CTLA-4 treated melanoma [97, 98]. Given that previous studies have proposed the importance of baseline derived NLR (dNLR) and LDH levels as prognostic markers, a recent study proposed a composite prognostic index that comprehensively takes the two factors into account, lung immune prognostic index (LIPI), which characterized 3 risk groups: good, intermediate, and poor [99]. The analysis of 3987 patients with advanced NSCLC in 11 randomized trials showed that patients with good LIPI score who received ICI were associated with significantly better PFS and OS compared with patients with poor LIPI score, which was not observed in patients received chemotherapy [99].

The study of melanoma treated with ipilimumab showed that the percentage of baseline CD45RO^+^/CD8^+^T cells was ≤25% in 80% of non-responders and ≥ 30% in all responders (*p* < 0.01) [100]. CyTOF analysis of melanoma treated with ICIs showed that the abundance of CD69^+^ and MIP1β^+^ NK cells [101] and CD14^+^CD16^−^HLA-DR^hi^ cells [102] were predictive biomarkers of response to anti-PD-1 therapy. In addition, ipilimumab treatment of melanoma with baseline high levels of circulating Tregs was associated with OS, possibly as a target for ipilimumab antibody ADCC due to its high CTLA-4 expression; whereas decreased or stabilized circulating Tregs at 12 weeks since ipilimumab initial administration was significantly associated with better DCR and OS [98]. Inducible T cell co-stimulator (ICOS) is costimulatory molecule expressed by activated T cells and Tregs. Analysis of surgical tissues and peripheral blood before and after treatment showed that anti-CTLA-4 treatment could induce ICOS pathway activation, and CD4^+^ICOS^+^T cells could produce IFN-γ and recognize tumor antigens [103]. In addition, a recent report correlated the detection of circulating tumor cells (CTCs) in peripheral blood with the metastatic process of tumors, and PD-L1 is highly expressed in CTCs from patients with advanced head and neck cancer, suggesting that PD-L1^+^CTC may be a predictive biomarker of response to ICIs [104].

### Biomarkers of circulating tumor DNA

The detection of circulating tumor DNA (ctDNA) can obtain tumor genomic information related to the response to ICIs, although the sensitivity or specificity has yet to be improved. Multiple studies showed that high mutation number of ctDNA was associated with improved OS and poor prognosis in patients with different cancer types treated with ICIs [105, 106]; Lee et al. [107] demonstrated that melanoma patients with persistently elevated ctDNA during PD-1 antibody therapy showed worse response and shorter PFS and OS. In addition, ctDNA can be a useful marker for identifying pseudoprogression during ICIs treatment. 9 patients with melanoma appeared pseudoprogression after ICIs therapy were reported to have favorable ctDNA profiles (defined as undetectable ctDNA or detectable ctDNA at baseline followed by > 10-times decrease in ctDNA), while 18 of 20 patients with true progression had unfavorable ctDNA profiles [108]. The association of bTMB level based on ctDNA and clinical benefit with anti-PD-1/L1 therapy was validated in tumor patients, confirming that it is a promising predictive biomarker. NCC-GP150 established using optimized gene panel size and algorithms is feasible for bTMB evaluation and bTMB can be used as a biomarker of clinical benefit in NSCLC patients treated with ICIs [109]. Another similar study showed that tTMB are strongly correlated (Spearman 0.6, Pearson 0.7) with bTMB evaluated with a 500-gene panel, which may serve as a potential biomarker for the efficacy of single and dual immunotherapy when cut-off value at 20 mt/Mb [110]. In addition, dynamic monitoring of ctDNA can provide ΔbTMB information to predict the responsiveness in the treatment process in a non-invasive manner, potentially improving the sensitivity and specificity of response prediction.

### Other circulating molecular biomarkers

Exosomes in the plasma can also provide information about the tumor and immunotherapy. Lower baseline levels and increases during treatment in circulating exosomal PD-L1 in melanoma patients were associated with response to pembrolizumab [111]. However, in another study of melanoma or NSCLS treated with anti-PD-1, the expression of PD-1 mRNA in the exosomes was higher at baseline and significantly decreased after treatment in patients with response, while it was stable in patients with stable disease and increased in patients with progressive disease after treatment [112]. Therefore, protein and transcripts of exosomal PD-L1 may provide contradictory information on the response to ICIs and require large-scale prospective studies for validation. In addition, RNA sequencing analysis of PD-L1 inhibitor-resistant NSCLC patients revealed the presence of PD-L1 variant fragments (v242 and v229, which retain the PD-1 binding domain) in vivo and in peripheral blood and pleural effusion, resistant patients with variant had much higher sPD-L1 concentrations. Experiments in vitro and vivo have confirmed the inhibitory effect of PD-L1 variant fragments on T cell activity [113], indicating a poor efficacy response.

In addition, other potential predictive biomarkers for ICIs efficacy have been preliminarily explored [114], including soluble proteins (e.g., sCD163, sNKG2DLs), cytokines and inflammatory factors [e.g., tumor necrosis factor (TNF)-α, interleukin (IL)-6, C-reactive protein (CRP)]. Baseline serum LDH is often an independent factor for poor prognosis and shorter OS with ipilimumab or pembrolizumab in patients with advanced melanoma [97, 115]. Several studies showed that in patients with various cancers treated with ICIs, high baseline LDH was associated with poor anti-tumor response [116, 117]. Weber et al. [118] analyzed baseline levels of CRP and IL-6 in serum from patients with melanoma who participated in 3 different clinical trials and levels above baseline median were found to be significantly associated with poor response and shorter survival after nivolumab treatment, and similar results were found with ipilimumab and combination therapy. In vitro studies revealed that purified CRP significantly inhibited T cell activation and proliferation at concentrations > 10 μg/mL [118]. Studies have also demonstrated that IL-6 has an immunosuppressive effect under certain conditions, including induction of myeloid-derived suppressor cells (MDSCs), which may explain the above phenomenon [119]. Besides, two retrospective studies involving approximately 3000 patients found that high baseline levels of plasma IL-8 were significantly associated with poor prognosis with PD-1/L1 inhibitors therapy and may be a driver of resistance to ICIs [120, 121]. scRNA-seq of the immune compartment showed that IL-8 is primarily expressed in circulating and intratumoral myeloid cells, and had an inhibitory effect on adaptive immunity. High IL-8 levels were associated with higher tumor neutrophil/monocyte infiltration, poorer antitumor activity of effector T cells, as well as weaker antigen presentation. Patients with both a higher T cell effect profile score and lower plasma IL-8 levels can obtain the greatest benefit from ICIs therapy.

(iv). **Host-related markers**

### General characteristics

Studies have shown that gender differences are associated with the responsiveness to anti-tumor immune. A meta-analysis including 20 randomized controlled trials (RCTs) of ICIs (*n* = 11,351) reported that gender difference in the efficacy ICIs was significant (*p* = 0.0019), with pooled OS hazard ratio being 0.72(95%CI 0.65–0.79) in male patients and 0.86(95%CI 0.79–0.93) in female patients [122]. In another meta-analysis of a large number of melanoma and NSCLC patients treated with ICIs, both PFS and OS were significantly longer in male patients than in female patients, and this difference was more pronounced in melanoma patients and anti-CTLA-4 antibodies [123]. Aging is associated with restricted immune function with significant effects on both innate and adaptive immune responses [124]. A preclinical study showed that aged mice had significantly increased tumor responses to anti-PD-1 agents compared with young mice, considered to be associated with a lower proportion of Tregs in aged mice [125]. Consistently, melanoma patients over 60 years old have a significantly higher tumor response to pembrolizumab than patients under 60, and the likelihood of response increases with age [125]. However, different results have also been reported by Nishijima et al. with an association between age less than 75 years and better ORR in patients treated with ICIs [126]. The Checkmate-171 trial showed that patients ≥70 years of age had comparable tolerability and efficacy to the overall population [127]. However, at present, the inclusion and representativeness of the elderly in clinical studies are still insufficient.

Besides, studies of the effect of performance status (PS) on the efficacy of ICIs have shown that good PS are associated with lower tumor burden and a predominance of immune cell function in TME. In the Checkmate-171, patients with PS =2 had inferior efficacy to the overall population [127], and real-world data in Israel again suggested that patients with PS ≥2 had inferior efficacy to the overall population [128]. A study reported by ASCO in 2018 (Justin F. et.al. 2018 ASCO abstract#9011) showed that the TMB of smoking patients with NSCLC in the two groups with equivalent PD-L1 expression was higher, and PFS and duration of response (DOR) in smoking patients with high PD-L1 expression (TPS ≥50%) were longer. Differences in body fat distribution also affects tumor prognosis and immunotherapy [129]. A study showed obesity induces T cell exhaustion and dysfunction by affecting PD-1 expression through STAT3 signaling, leading to increased immune aging and promoting tumor growth and progression [130]. However, targeting receptors on activated T cells or chimeric antigen receptor (CAR)-T cells in immunotherapy may help enhance T cell function, especially in the presence of high leptin. Studies have found that obese mice also showed a significantly better response to anti-PD-1 without significant toxic side effects [130], which was reproduced in multiple cancer populations receiving ICIs [131], with higher body mass index (BMI) (BMI > 30) patients showing reduction in tumor burden and improvement in PFS and OS. Although the mechanisms by which baseline general characteristics influence the efficacy of ICIs have not been fully demonstrated, they can be used as stratification factors for efficacy and tumor prognosis in future trials to gradually expand the understanding.

### Intestinal commensal microbiota

The commensal microbiota plays a key role in the immune response, with gut bacteria significantly associated with improved responses to ICIs in humans [132]. Four independent studies analyzing baseline fecal samples found that different specific intestinal bacterium were associated with response to ICIs in melanoma [133–135], NSCLC, RCC, and urothelial carcinoma [132]; Sivan et al. [136] reported that commensal bifidobacteria enhanced anti-PD-1 antibody response by enhancing DC function in mice; PFS and OS after ipilimumab treatment in melanoma patients with baseline microbiota enriched Faecalibacterium species and other Firmicutes were better than those with baseline microbiota enriched Bacteroides [134]; in addition, Routy et al. [132] revealed a correlation between clinical response and the relative abundance of Akkermansia muciniphilia, which enhanced the efficacy of PD-1 antibodies in an IL-12-dependent manner. The impact on the efficacy of ICIs may be related to different cancer types, microbial sequencing and analysis techniques, geographical distribution of intestinal bacteria, as well as antibiotic treatment.

### Host germline genetics

HLA genes are the most polymorphic genes in the human genome and encode key components of immunogenicity. HLA-I diversity is characterized by significant sequence variation in peptide-binding regions, termed the human immunopeptidome [137]. Analysis of 1535 patients with ICI-treated tumors found that the presence of a more diverse array of HLA-I molecules was associated with increased survival [138], possibly due to its broader presentation of tumor antigens [139, 140]. Patients who are heterozygous for all HLA-I loci in patients receiving anti-PD-1 therapy have a higher on-treatment clonal expansion of TCR repertoire than homozygous patients [139]. A study showed that HLA loss of heterozygosity (HLA LOH) occurs in 45.1% of all patients with advanced disease and varies by tumor type [14]. The concordance of HLA LOH detected by WES and multi-genic panels is high and suggests that HLA LOH may be associated with immune escape, resulting in the resistance to immunotherapy [141]. In addition, specific HLA-I supertypes, such as the HLAB44 supertype allele, are associated with improved survival in melanoma patients treated with ICIs [139]. Other host immune-related gene polymorphisms, including HLA-II genes, non-classical HLA-i genes, NF-κB, and JAK-STAT family members, have also been shown to be associated with tumor response to ICIs [138]. Future studies are needed to further investigate the impact of host immune gene variation on ICIs efficacy.

(v). **Immune-related adverse events**

Since ICIs may cause tumor regression and immune-related adverse events (irAEs) through enhanced immune responses, several studies have shown a relevance of the two. irAEs are associated with tumor regression in patients with metastatic RCC or melanoma treated with ipilimumab [142, 143]. And the early development of overall irAEs was associated with better ORR and PFS in NSCLC patients treated with nivolumab [144]. However, multivariate analysis by Judo [145] showed that only low-grade irAEs, but not high-grade irAEs, were associated with better response to anti-PD-1 blockade in patients with non-melanoma. In addition, different types of irAEs are associated with immunotherapeutic responses in different tumor types. Fujisawa et al. [146] demonstrated an association between endocrine irAEs and OS and better prognosis in melanoma patients treated with ipilimumab after nivolumab; likewise, thyroid dysfunction in NSCLC patients treated with anti-PD-1 was statistically associated with OS and PFS [147]. The development of vitiligo is associated with a better response to ipilimumab in melanoma patients [148], may representing a common immune response against antigens shared by melanocytes and melanomas. Several studies showed that other skin irAEs were also associated with better outcomes in cancer [149, 150]. But the controversial finding reported that the occurrence of skin toxicities, except for vitiligo, were related to a shorter OS of melanoma patients treated with ipilimumab after nivolumab [146]. Since skin irAEs include various types of skin disorders, such as pruritus, psoriasis, and lichenoid toxicity, the association of each skin irAE with outcome may vary. However, given that ICIs may cause tumor regression and irAEs by enhancing immune responses, some biomarkers that have been explored to predict the occurrence of irAEs, such as T cell diversity, cytokines and inflammatory factors, different gut microbiome, may also be predictive biomarkers of ICIs-efficacy. Therefore, in clinical practice, how to better use biomarkers to achieve the best efficacy while experienced minimal toxicity is a difficult problem to explore in the future.

**Fig. 1:**
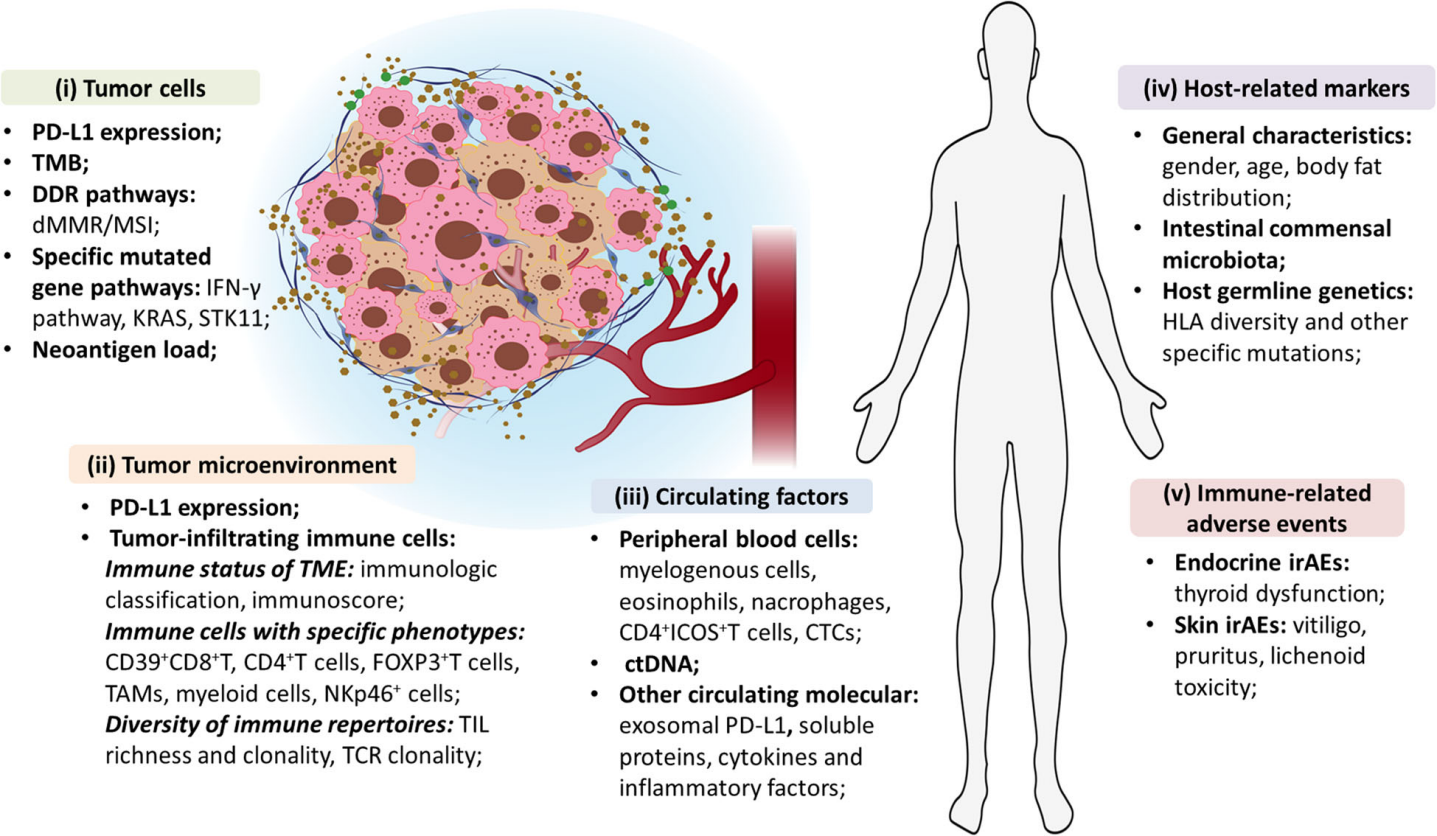
An overview of predictive biomarkers for immune checkpoint inhibitors efficacy. Key elements in predictive biomarker development for the efficacy of immune checkpoint inhibitors therapy are briefly described in the figure, including tumor cells-related biomarkers, tumor immune microenvironment phenotype biomarkers, circulating factors, host-related factors, and immune-related adverse events

There provides an overview of predictive biomarkers for ICIs efficacy in Fig. 1, and details of some factors in Table 1.

**Table 1:** Details of some factors that predict response to immune checkpoint inhibitor therapy

Type of marker	Marker	Association with clinical outcome	Cancer type	Tissue type for marker assessment
**Tumor genome and neoantigen biomarkers**	Tumor mutation burden	Positive or negative	Multiple tumor types	Tumor tissue or blood
	PD-L1 expression in tumor	Positive	Multiple tumor types	Tumor tissue
	Iindel	Positive	Multiple tumor types	Tumor tissue
	SCNAs	Positive	Multiple tumor types	Tumor tissue
	DNAm, e.g., HOX gene methylation	Positive	NSCLC	Tumor tissue or blood
	DDR pathways, e.g., dMMR/MSI, BER, HRR;	Positive	Multiple tumor types	Tumor tissue
	IFN-γ pathway genes, IFNGR1/2, JAK1/2, and IRF1	Negative	Multiple tumor types	Tumor tissue or blood
	STK11	Positive or Unknown	NSCLC	Tumor tissue or blood
	Neoantigen load, low neoantigen intratumour heterogeneity	Positive	Multiple tumor type	Tumor tissue
**Tumor immune microenvironment phenotype biomarkers**	PD-L1 expression in TME	Positive	Multiple tumor types	Tumor tissue
	Immune-inflamed TME	Positive	Multiple tumor types	Tumor tissue
	T cell repertoire clonality	Positive	Multiple tumor types	Tumor tissue or blood
	CD39 + CD8 + TIL	Positive	NSCLC, RCC	Tumor tissue or blood
**Liquid biopsy biomarkers**	NLR	Negative	Melanoma, NSCLC	Blood
	High mutation number of ctDNA or favorable ctDNA profiles	Positive	Multiple tumor types	Blood
	LDH	Negative	Melanoma	Blood
	IL-8	Negative	Multiple tumor types	Blood
	Exosomal PD-L1	Positive or negative	Melanoma, NSCLC	Blood
	PD-L1 variant fragments	Negative	NSCLC	Blood
**Host-related markers**	Gender	Male: positive	Multiple tumor types	–
	Age	Positive or negative or Unknown	Multiple tumor types	–
	Body fat distribution	Positive	Multiple tumor types	–
	Specific Intestinal microbiota	Positive or negative	Multiple tumor types	Oral or gut
	HLA-I diversity	Positive	Melanoma, NSCLC	Blood
	HLA LOH	Negative	Melanoma	Tumor tissue
**irAEs**	irAEs in different organs	Positive or Unknown	Multiple tumor types	–

*PD-L1* programmed cell death-ligand 1, *RCC* renal cell carcinoma, *NSCLC* non-small cell lung cancer, *TMB* tumor mutation burden, *indel* insertion and deletion, *SCNAs* somatic copy number alterations, *MMR* mismatch repair, *dMMR* MMR deficiency, *MSI* microsatellite instability, *TIL* tumor infiltrating lymphocyte, *POLE/POLD1* polymerase gene epsilon/delta 1, *BER* base excision repair, *HRR* homologous recombination repair, *DDR* DNA damage response, *HLA* human leukocyte antigen, *TME* tumor microenvironment, *NLR* neutrophil-to-lymphocyte ratio, *ctDNA* circulating tumor DNA, *IL-8* interleukin-8, *LDH* lactate dehydrogenase, *irAE* immune-related adverse event, *DNAm* DNA methylation *HLA LOH* HLA loss of heterozygosity

## Exploration of predictive markers by ICI types

In addition, considering that the type of ICIs is more correlated with treatment, it seems more reasonable to explore biomarkers that can predict the efficacy of different ICIs. Studies have shown an association between tumor autoantigen expression and improved ICI-response. Eight-gene cluster known as the “anti-CTLA-4 resistance associated MAGE-A (CRMA)” cluster is associated with poor response to anti-CTLA-4 rather than anti-PD-1 therapy [151]. The exact mechanism is unknown, but may be related to the idea that the expression of CRMA leads to a reduction or defect in autophagy, which in turn interferes with antigen processing and presentation. Therefore, CRMA expression is considered to be a predictive biomarker for anti-CTLA-4 therapy rather than a predictor of overall disease prognosis, and CRMA gene expression may be used to identify patients who respond to combination therapy of anti-CTLA-4 and anti-PD-1 [151]. The researchers analyzed the expression MHC-I and II protein in tumor cells from previously untreated patients with advanced melanoma, and correlated the results with transcriptomic and genomics analyses [152]. They found that MHC proteins showed different sensitivities to CTLA-4 and PD-1 blockers. Major (> 50%) or complete loss of MHC-I expression on membranes of melanoma cell was associated with transcriptional repression of HLA-A, HLA-B, HLA-C, and B2M in 78/181 patients (43%), which could predict the resistance to anti-CTLA-4 antibody therapy but not anti-PD-1 therapy. MHC-II expression was observed in > 1% of melanoma cells in 55/181 (30%) patients, and correlated with IFN-γ and its mediated gene signature, which could predict the response to anti-PD-1 but not anti-CTLA-4 therapy [152]. Thus, MHC-I expression is required for the primary response against CTLA-4 for melanoma, while the primary response to anti-PD-1 is associated with pre-existing IFN-γ-mediated immune activation. Therefore, the exploration of markers to predict the therapeutic efficacy or resistance of different ICIs is also essential. More studies are expected in the future to analyze the mechanisms of action of different ICIs and their interactions with tumors in depth.

## Comprehensive predictors of ICIs efficacy

The current understanding of the clinical response to ICIs-treatment suggests that any single biomarker cannot effectively identify the benefit populations. The specificity and efficacy of prediction will be greatly improved when combination of multiple factors is used as a composite variable to capture immune status. Rizvi et al. [153] found that TMB and PD-L1 were two independent factors affecting the efficacy of immunotherapy, while patients with both high levels of TMB and positive PD-L1 had the highest duration of benefit rate; another study showed that NSCLC patients with both high TIL density and high PD-L1 expression treated with PD-L1 inhibitor had the highest positive predictive value of ORR and the longest PFS [154]; and Yu et al. [155] further demonstrated that the comprehensive variables of three predictive markers, CD8^+^TIL, PD-L1 expression, and TMB, were associated with improved OS and PFS compared with a single biomarker or two of the three biomarkers. Furthermore, the use of big data analysis to predict markers of immunotherapy efficacy helps to establish a new framework for precise treatment of tumors. A study of 4 groups of clinical trials covering 22 cancer types and more than 300 patients evaluated the relationship between biomarkers and best overall responses (BOR), PFS. It was found that TMB, T cell-inflamed GEPs were associated with the efficacy of clinical immunotherapy, and the higher TMB, the higher ORR [156].

In addition, developing predictive models by integrating different types of data based on different components of tumor-host interactions seems to have a good prospect. A research team created two neoantigen immune fitness models by computational biology methods, namely, the neoantigen quantity model, mainly statistically analyzing the number of tumor antigens, and the neoantigen quality fitness model, involving various factors such as the similarity between tumor antigens and pathogen antigens and the binding ability to TCR [157]. The results showed that only the neoantigen quality fitness model could better predict the postoperative survival of patients with pancreatic cancer. Another study developed a new neoantigen fitness model including three elements (tumor clonality, DAI, and microbial epitope homology), which was quantified as a nonlinear function of alignment scores, and the results showed that the model incorporating all three elements successfully predicted survival in all three ICI-treatment cohorts [60]. But before applying the model more broadly, it is necessary to identify unique parameters for each cancer species and/or therapeutic agent [158]. Jiang et al. [159] designed a completely new computational architecture, TIDE score ratio biomarkers (tumor mutation load, PD-L1 level, and INF-γ), namely tumor immune dysfunction and rejection scores. It reveals the impact of tumor infiltration levels of different immune cell types on overall survival of patient by analyzing the TCGA and PRECOG databases and synthesizing different types of tumor immune escape mechanisms. Using this framework and pretreatment RNA-Seq or NanoString tumor expression profiling, they have identified that TIDE more accurately predicts the outcome of melanoma patients treated with first-line anti-PD-1 or anti-CTLA-4 than other biomarkers such as PD-L1 levels and TMB. TIDE also revealed novel candidate regulators of resistance to ICIs, such as SERPINB9, demonstrating utility for immunotherapeutic studies.

The combined detection of independent predictive markers makes more patients to receive ICIs and expands the beneficiary population, while for interacting markers, a bioinformatics-based predictive-model can be established according to different impact weights of each factor and improve the accuracy of screening the beneficiary population by comprehensive consideration, and how to better utilize the interrelationship network of various markers is an aspect to be considered of comprehensive predictive-models; in addition, it should be explored how the combined prediction with multiple factors achieve the optimal cost-effectiveness to serve the clinical immunotherapy of tumors more effectively. In the future, it may be promising to obtain the most effectively comprehensive predictive-markers by extracting features with large samples and multiple dimensions and constructing multivariate models using machine learning and artificial intelligence.

## Summary and outlook

In this review, we deeply analyze the exploration course and research progress of predictive biomarkers as an adjunctive tool to tumor immunotherapy in identifying ICIs efficacy. In recent years, predictive markers of ICIs efficacy have been gradually explored from the expression of intermolecular interactions within tumor cells to the expression of various molecules and cells in TME, even been extended to the exploration of circulating and host systemic markers, and gradually realized the process from the identification of single marker to the development of multifactorial synergistic predictive markers. The exploration of predictive biomarkers of ICIs efficacy indicates a complex interaction between the regulation of the immune system network and tumors, reflecting more comprehensively the complexity and diversity of the effects of immunotherapy on tumors and even the whole body. Nonetheless, the findings of some biomarkers explored in the review are contradictory and the mechanisms of action are not well understood, which need to be confirmed by further large-scale prospective studies, but these breakthrough findings offer a great promise for biomarker strategies with more accurate positive and negative predictive values that can be used routinely in clinical practice to assist patients with different malignancies in ICIs-based therapy management, monitor disease development, and conquer tumor resistant to immunotherapy.

With the development of basic technology research such as multiplex IHC, high-throughput sequencing technology, and microarray technology, more and more potential markers can be widely screened widely on a genomic scale and a variety of proteins and cell populations can be quantified. However, several knowledge gaps still exist. First, for single marker, further cognition of PD-L1 and TMB should be enhanced, while continuing to promote consistency evaluation of detection methods; Gene mutations show great potential in the evaluation and monitoring of the whole course of immunotherapy and should be continuously explored; There are still many unknowns about the exploration of markers of the immune microenvironment and host microenvironment, which needs to be understood from a deeper molecular perspective. Secondly, in view of the various emerging biomarkers and the disadvantages of every single marker to varying degrees, strategies combining two or more approaches to capture immune status may be more effective as composite predictive biomarkers for ICIs efficacy. The advantages of each marker should be fully utilized to lay the foundation for the development of multifactorial predictive models. Balancing the relationship between the scientificity, accessibility, and simple operation of the clinical application of each predictive marker/model is a challenge to consider in clinical research. Thirdly, the exploration of more simple and feasible prediction means in clinical practice. For example, the potential of liquid biopsy such as ctDNA in the whole process of efficacy evaluation and monitoring of immunotherapy should be fully developed. Predicting the long-term survival of immunotherapy based on biomarkers in peripheral blood is a potential development direction. Furthermore, the use of machine deep learning and artificial intelligence to explore the mechanisms and markers of immunotherapy efficacy and drug resistance is changing from fantasy to reality, which can be used as the direction of future scientific research and clinical exploration. Multivariate predictive models need to extract data features with large samples and multiple dimensions using machine learning, and integrate different types of data based on different components of tumor-host interactions for comprehensive validation and evaluation, including polymorphism data such as intratumoral genomic and molecular characteristics, tumor immune microenvironment phenotype, peripheral blood biomarkers and host-related factors. Finally, given that multiple patterns of atypical response, such as pseudoprogression, occur during immunotherapy, which significantly affect patient treatment and overall survival, it is also essential for the exploration of predictive markers to these special response pattern. In the future, through the scientific study of the availability of multiple markers and the exploration of feasibility and reproducibility in clinical practice, standardized predictive biomarkers (models) for ICIs response would be established to maximize the benefit of patients from these transformative treatments, ultimately prompting the field to develop towards precision immuno-oncology.

## Data Availability

Not applicable.

## Abbreviations

CAR: chimeric antigen receptor
BOR: best overall responses
ICI: Immune checkpoint inhibitor
PD-1/PD-L1: Programmed cell death-1/programmed cell death-ligand 1
RCC: Renal cell carcinoma
HNSCC: Head and neck squamous cell carcinoma
ORR: Objective response rate
NSCLC: Non-small cell lung cancer
TMB: Tumor mutation burden
SCLC: Small cell lung cancer
HPV: Human papilloma virus
NCCN: National Comprehensive Cancer Network
OS: Overall survival
DCR: Disease control rate
DCB: Durable clinical benefit
PFS: Progression-free survival
CGP: Comprehensive genomic profiling
NGS: Next generation sequencing
AF: Allele frequency
CNV: Copy number variation
nsSNV: Nonsynonymous single nucleotide variant
SCNAs: Somatic copy number alterations
indel: Insertion and deletion
TCGA: The Cancer Genome Atlas
MMR: Mismatch repair
dMMR: MMR deficiency
MSI: Microsatellite instability
TIL: Tumor infiltrating lymphocyte
IDO: Indoleamine 2,3-dioxygenase
POLE/POLD1: Polymerase gene epsilon/delta 1
BER: Base excision repair
HRR: Homologous recombination repair
DDR: DNA damage response
dsRNA: Double-stranded RNA
ADAR1: Adenosine deaminase acting on RNA
MDA5: Melanoma differentiation-associated protein 5
CTLA-4: Cytotoxic T-lymphocyte-associated protein-4
EGFR: Epidermal growth factor receptor
ALK: Anaplastic lymphoma kinase
FDA: Food and Drug Administration
AACR: American Association for Cancer Research
ERV: Endogenous retroviruse
MHC: Major histocompatibility complex
DAI: Differential agretopicity index
IEDB: Immune Epitope Database
HLA: Human leukocyte antigen
IHC: Immunohistochemistry
TPS: Tumor proportion score
CPS: Combined positive score
IPS: Immune positive score
TIM-3: T cell immunoglobulin-3
LAG-3: Lymphocyte activation gene-3
VISTA: V-domain Ig suppressor of T-cell activation
TME: Tumor microenvironment
ROC curve: Receiver operating characteristic curve
scRNA-seq: Single-cell mRNA sequencing
TCF7: Transcription factor 7
FcγR: Fc domain glycan of the drug and Fcγ receptor
TAM: Tumor-associated macrophage
NK cell: Natural killer cell
TCR: T cell receptor
IR: Immune repertoire
GEP: Gene expression profile
NLR: Neutrophil-to-lymphocyte ratio
ICOS: Inducible T Cell Co-stimulator
CTC: Circulating tumor cell
ctDNA: Circulating tumor DNA
MDSC: Myeloid-derived suppressor cell
TNF: Tumor necrosis factor
IL: Interleukin
CRP: C-reactive protein
LDH: Lactate dehydrogenase
RCT: Randomized controlled trial
PS: Performance status
ASCO: American Society of Clinical Oncology
DOR: Duration of response
BMI: Body mass index
irAE: immune-related adverse event
WES: whole exome sequencing
HLA LOH: HLA loss of heterozygosity
dNLR: derived NLR
LIPI: lung immune prognostic index
FGFR2: fibroblast growth factor receptor 2
METex14: MET exon 14 skipping
CRMA: CTLA-4 resistance associated MAGE-A
